# Current Strategies for the Manufacture of Small Size Tissue Engineering Vascular Grafts

**DOI:** 10.3389/fbioe.2018.00041

**Published:** 2018-04-17

**Authors:** Michele Carrabba, Paolo Madeddu

**Affiliations:** School of Clinical Sciences, Bristol Heart Institute, University of Bristol, Bristol, United Kingdom

**Keywords:** tissue engineering, vascular conduits, myocardial ischemia, regenerative medicine, stem cells

## Abstract

Occlusive arterial disease, including coronary heart disease (CHD) and peripheral arterial disease (PAD), is the main cause of death, with an annual mortality incidence predicted to rise to 23.3 million worldwide by 2030. Current revascularization techniques consist of angioplasty, placement of a stent, or surgical bypass grafting. Autologous vessels, such as the saphenous vein and internal thoracic artery, represent the gold standard grafts for small-diameter vessels. However, they require invasive harvesting and are often unavailable. Synthetic vascular grafts represent an alternative to autologous vessels. These grafts have shown satisfactory long-term results for replacement of large- and medium-diameter arteries, such as the carotid or common femoral artery, but have poor patency rates when applied to small-diameter vessels, such as coronary arteries and arteries below the knee. Considering the limitations of current vascular bypass conduits, a tissue-engineered vascular graft (TEVG) with the ability to grow, remodel, and repair *in vivo* presents a potential solution for the future of vascular surgery. Here, we review the different methods that research groups have been investigating to create TEVGs in the last decades. We focus on the techniques employed in the manufacturing process of the grafts and categorize the approaches as scaffold-based (synthetic, natural, or hybrid) or self-assembled (cell-sheet, microtissue aggregation and bioprinting). Moreover, we highlight the attempts made so far to translate this new strategy from the bench to the bedside.

## Introduction

Cardiovascular disease is the principal cause of death worldwide. Common manifestations are coronary heart disease (CHD) and peripheral arterial disease (PAD), which develop as a consequence of the critical atherosclerotic narrowing of supplying arteries. The worldwide annual incidence of deaths related to cardiovascular disease, is expected to rise to 23.3 million by 2030 (Criqui and Aboyans, 2015). In the UK alone, more than 2 million people suffer from CHD. Moreover, there are 188,000 hospital episodes attributed to a myocardial infarction (MI) each year. Approximately 12 to 20% of people over the age of 60 develop PAD and many of them manifest critical limb ischemia, which is associated with a poor quality of life and high risk of amputation and death (Townsend et al., 2015).

Prompt restoration of tissue perfusion is pivotal for preventing heart failure in patients with CHD and for helping the repair of ischemic limbs. Current revascularization techniques consist of angioplasty, placement of a stent, or surgical bypass grafting. In the United States, an average of 400,000 coronary artery bypass grafting (CABG) interventions are recorded annually (Epstein et al., 2011; Pashneh-Tala et al., 2015). Moreover, the number of invasive lower-extremity vascular procedures for patients with PAD has doubled over the last decade. The surgical bypass grafting mostly involves the use of autologous vessels, such as the saphenous vein and internal thoracic artery. Though representing the gold standard for small-diameter (<6mm) vascular replacement, these vessels require invasive harvesting and are often unsuitable for use. For instance, in patients needing primary revascularization of the lower extremities, as many as 30% lack a suitable autogenous vein. This number increases to 50% in those patients requiring a secondary bypass procedure. Furthermore, venous grafts can develop neointimal hyperplasia in the peri-anastomotic regions. Patency rates for saphenous vein grafting remain limited, with both coronary and femoro-popliteal reconstructions showing failure rates of approximately 50% at 10 years (Goldman et al., 2004; van Dijk et al., 2007; Collins et al., 2008; Kim et al., 2008; Schwann et al., 2009; Harskamp et al., 2013).

Surgical revascularization with implantation of conduits made of non-biodegradable polymers, including Polytetrafluoroethylene (PTFE), Gore-Tex, and Dacron, prove to be effective when replacing large vessels. However, when used in the application of small-diameter vascular grafts, they were complicated by the occurrence of thrombotic occlusions (Jackson et al., 2000; van Det et al., 2009; Takagi et al., 2010).

Considering the limitations of current vascular bypass conduits (Gui and Niklason, 2014), a tissue-engineered vascular graft (TEVG) embedded with cells to generate a living material capable of physiological remodeling represents a potential solution for the future of vascular surgery. In this review, we provide a summary of methodologies and solutions adopted in recent years that aim to create functional small-diameter TEVGs.

## Tissue engineering

Halfway through the twentieth century, the first autologous saphenous vein was used as a vascular graft in a clinical application by Kunlin (1951). By the end of the 1970s, synthetic material such as Dacron (De Bakey et al., 1958) and PTFE (Soyer et al., 1972; Campbell et al., 1976; Tellis et al., 1979), were introduced for aortic and lower extremity bypass, respectively. As mentioned above, the low patency rate over a long period of implantation was the common limitation of these vascular grafts.

Tissue engineering aims to provide alternative and innovative solutions for small diameter vascular replacement, and an interdisciplinary approach could offer the chance to design a graft for any specific target tissue and clinical needs.

The first commercially available acellular TEVG from bovine and human origin, such as Artegraft (North Brunswick, NJ) (Hutchin et al., 1975), Procol (Hancock Jaffe Laboratories Inc., Irvine, CA) (Hatzibaloglou et al., 2004), and Cryovein (CryoLife, Kennesaw, GA) (Madden et al., 2004), appeared on the market toward the end of the 1970s. Nevertheless, the flourishing number of techniques and innovative approaches finds its definitive turning point with Weinberg and Bell in 1986, who first tried to fabricate a biological vascular graft with xenogenic cells embedded to circumvent the limited availability of autologous cells (Weinberg and Bell, 1986). A collagen gel was used as a substrate on which they cultured bovine fibroblasts, vascular smooth muscle cells (VSMCs) and endothelial cells (ECs), thereby recreating the adventitia, media and intima layers of the vessel, respectively. This attempt led to the fabrication of a vessel-like structure with poor mechanical properties, that required a Dacron mesh to act as a structural support. Despite its apparent failure, this pioneering study drew a new path in TEVG development. From then on, many attempts have been made, ultimately leading to a standardized set of quality control criteria for TEVG fabrication, that is based on the performance of the actual “gold standard” [for example the saphenous vein (SV) or internal mammary artery (IMA)]. The ideal TEVG, therefore, should have anti-thrombogenic properties preferentially conferred by a fully autologous endothelium. Another important quality requisite is the similarity of TEVG mechanical properties to the native tissue, with a recommended minimum burst pressure of 1700 mmHg (Konig et al., 2010; Wise et al., 2011), together with a fatigue resistance of 30 days to cyclic loading *in vitro* (L'Heureux et al., 2007) and a level of compliance necessary to avoid excessive stress. A mechanical mismatch is acknowledged as a key determinant in the loss of long-term patency, resulting in aneurysm formation and implant at failure. The living cell component within the TEVG is critical to provide remodeling potential and biochemical signaling (G et al., 2015) while being devoid of immunogenic activity. To achieve clinically valuable outcomes, the manufacturing process has to take into account other fundamental aspects, such as the capability of the TEVG to be stored and delivered ready for the intervention as well as to be easily manipulated during the implantation.

TEVGs can be mainly categorized into self-assembled vascular grafts and scaffold-based approaches, using synthetic, natural or hybrid materials. Natural polymers, can then be further categorized into extracellular matrix (ECM)-based material and decellularized natural matrices.

### Scaffold-based TEVGs

The scaffold-based approach represents the most diffuse strategy to build TEVGs. The popularity of this methodology is justified by the fact that the presence of physical support enables the cells to follow a pathway during their colonization and proliferation. As introduced previously, the study performed by Weinberg and Bell pioneered the development of the scaffold-based methodology which during the last 30 years, saw the introduction of many variables and the use of a great variety of manufacturing techniques and materials.

#### Synthetic materials

Synthetic polymers have been widely used for the fabrication of TEVGs. The advantage is that the final properties of the graft can be tuned to meet the clinical needs, choosing the appropriate fabrication technique and specific material. However, the required higher level of technologies and the long period involved in the process of manufacturing constitute significant obstacles to clinical translation. Other prominant disadvantages shown by these materials are the lack of cell binding sites and the necessity to ensure an anti-thrombogenic property of the lumen, as required in the case of PLGA. A variety of polymers and copolymers have been tested. The most studied comprise degradable polyesters, like polyglycolic acid (PGA) (Niklason and Langer, 1997; Hoerstrup et al., 2006), poly-lactic acid (PLA), poly-l-lactic acid (PLLA) (Yokota et al., 2008), their copolymer poly (lactide-co-glycolide) (PLGA) (In Jeong et al., 2007), and polycaprolactone. Among the biodegradable polymers, polyurethanes (PU) (Hashi et al., 2007; Nieponice et al., 2010; Sharifpoor et al., 2011) and Poly(glycerol-sebacate) (PGS) (Wu et al., 2012), which are bioabsorbable elastomers, possess good biocompatibility properties allowing proliferation of endothelial cells (ECs) onto the luminal side and parietal infiltration of VSMCs (Gao et al., 2006; Rai et al., 2012). Hemocompatibility testing sees PGS having a low platelet adhesion and inflammation (Motlagh et al., 2006) and stimulating the production of elastin (Lee et al., 2011). However, the main issue was represented by the lack of mechanical properties, with a burst pressure of 200 mmHg (Lee et al., 2011). Various TEVG models fabricated with synthetic polymers have been assessed in preclinical small and large animal models. Both PU (Nieponice et al., 2010) seeded with mouse-derived MSCs and bone marrow-derived stem cells (BMDSCs) seeded onto PLA (Hashi et al., 2007) vascular grafts were used in a rat model and showed patency rates of 50 and 100% respectively, after several weeks from implantation. PGA is the most extensively explored material having been used in sheep (Brennan et al., 2008; Cummings et al., 2012), dogs, pigs (Quint et al., 2011) and primate models. Acellular electrospun PCL conduits, implanted into a mouse carotid model, allowed complete endothelium formation in 28 days. However, neointimal formation was detected, in particular at the anastomoses (Chan et al., 2017). Electrospinning microfabrication technique is often used to generate tubular structures composed of nanofibers from different polymers. Composite scaffolds made of PCL/poly(ethylene oxide) (Wang et al., 2016) and PCL/PLGA (Ong et al., 2017) have been tested in animal models with the later acellular graft having been evaluated in an ovine bilateral arteriovenous shunt model. Such a model showed good results in term of patency (66%) and endothelialization of the lumen after 4 weeks of implantation, but the graft eventually dilated as a consequence of inadequate elastin content (Ong et al., 2017).

Despite the great variety of preclinical studies, very few assessed the clinical utility of this type of TEVGs. One key study involving synthetic biodegradable polymers has been carried out by Niklason et al. using PGA seeded with VSMCs to create a small diameter TEVG (Niklason and Langer, 1997). The graft was conditioned with pulsatile flow for 8 weeks to achieve full maturation, through deposition of collagen matrix. At the end of the *in vitro* culturing, the structure showed a burst pressure of 2150 mmHg (Niklason, 1999; Niklason et al., 2001). These engineered blood vessels, named Humacyte (Humacyte Incorporated, RTP, NC), were tested in a series of small and large animals models, showing 100% patency after 24 days and 88% after 6 months in dogs and baboons, respectively (Dahl et al., 2011, 2013). These promising results led to a clinical trial that got underway in 2012, in which the acellular PGA scaffolds were used for vascular access in patients with end-stage renal disease (Gui and Niklason, 2014; Lawson et al., 2016). This study involved 60 patients recruited in Poland and the US with an average of follow-up period of 16 months, during which 4 patients died, although none were associated with the failure of the graft (Lawson et al., 2016) (Table 1). Additionally no immune response or aneurysm formation was detected. In term of efficacy, the TEVGs were successfully patent (63%) at 6 months, while the patency rate dropped to 28% after 12 months. This led to numerous interventions of thrombectomy to restore the patency (Lawson et al., 2016).

**Table 1:** TEVG applied in human studies.

**Approaches**	**Applications**	**Scaffold materials and fabrication methods**	**Groups**
Natural material-based TEVG	Graft as an extrahepatic portal vein bypass.	Decellularized human iliac vein seeded with autologous cells **No commercial product**	Sumitran-Holgersson and colleagues (Olausson et al., 2012)
	Lower extremity bypass surgery	Decellularized bovine carotid artery graft **Commercial name**: Artegraft, (North Brunswick, NJ)	Lin and colleagues (Lindsey et al., 2017)
Synthetic material-based TEVG	Arteriovenous (AV) shunt for heamodyalisis	Decellularization of PGA scaffolds seeded with cadaver SMCs. **Commercial name**: Humacyte, (Humacyte Incorporated, RTP, NC)	Dahl/Niklason and colleagues (Lawson et al., 2016)
Self-assembled TEVG	AV shunt for heamodyalisis access	Cell-sheet of human fibroblast in a shape of conduit. ECs were seeded in the graft after devitalization of the luminal side. **Commercial name**: Cytograft, (Cytograft Tissue Engineering, Inc.)	L'Heureux and colleagues (Wystrychowski et al., 2011)
	AV shunts for hemodialysis access	Cell-sheet of human fibroblast in a shape of conduit, without further endothelialization. Dehydrated and stored (−80°C) before clinical application. **Commercial name**: LifeLine™, (Cytograft Tissue Engineering, Inc.)	L'Heureux and colleagues (Wystrychowski et al., 2014)

#### Natural materials

##### ECM based grafts

**Figure 1:**
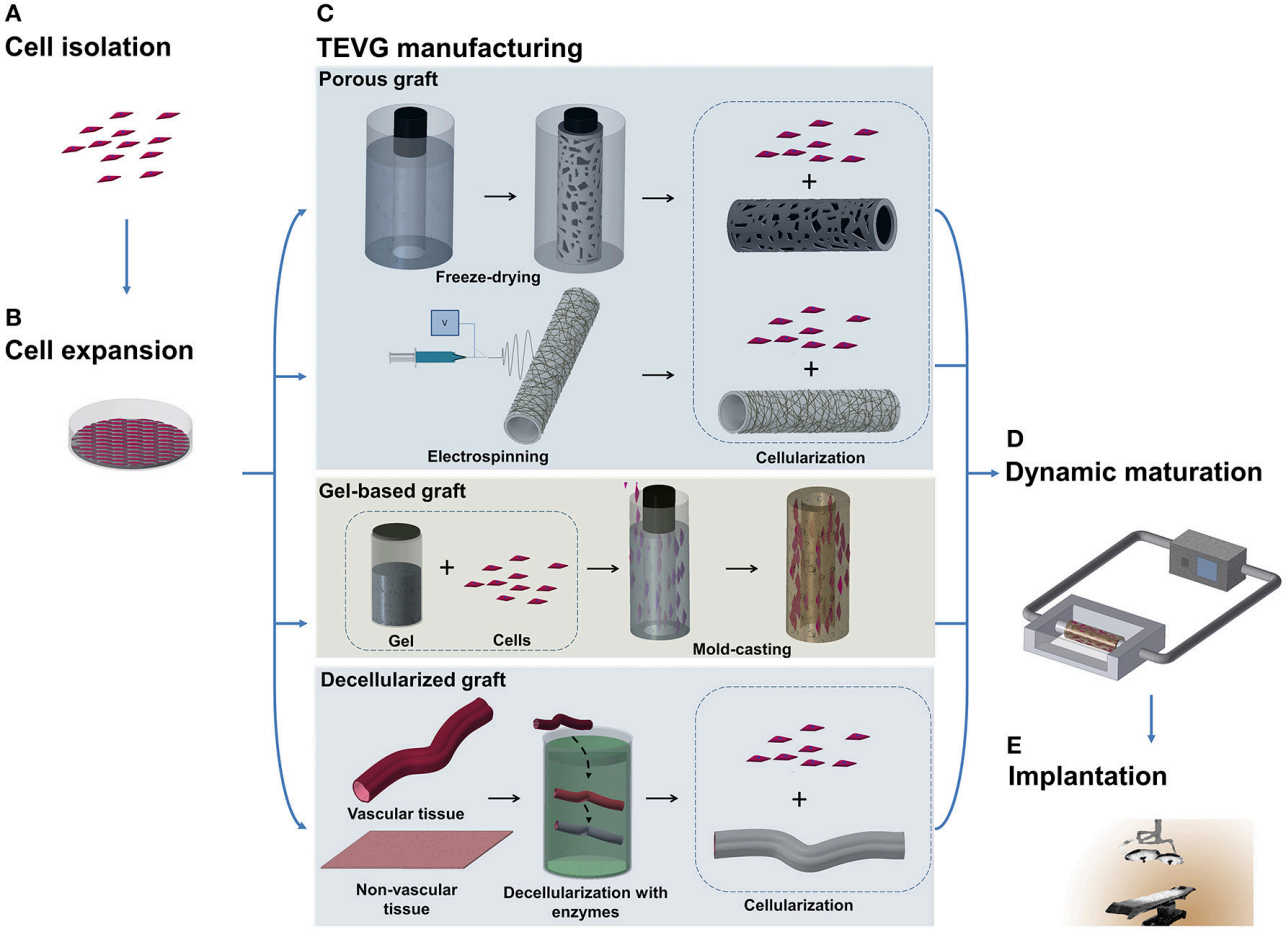
Schematic illustration of TEVG manufacturing process. **(A,B)** Tissues obtained from biopsies of patients are treated and cells are isolated and expanded *in vitro*. **(C)** Microfabrication techniques, such as freeze-drying and electrospinning, can be used to treat natural and synthetic materials in order to obtain a porous scaffold. Another approach sees the casting of a suspension of gel and cells into a mold to produce a tubular structure. Vascular and non-vascular tissues, obtained from allogeneic or xenogeneic sources, are used as TEVGs after being decellularized by using detergents and enzymes. **(D)** The TEVG undergoes cellularization with the expanded autologous cells before moving to dynamic conditioning into bioreactor which allows the maturation of the structure. **(E)** The manufactured TEVG is implanted into the patient.

The lack of bio-activity of synthetic scaffolds prompted researchers to investigate natural polymers obtained from ECM as a possible alternative option. Proteins derived from the ECM have the benefit of maintaining the natural binding sites for cell adhesion, improving biomimetic and biocompatibility properties of the material and stimulating the colonization and proliferation of recruited cells. Collagen, gelatin, elastin, fibrin, and silk-fibroin are the most extensively used in tissue engineering. Different manufacturing techniques can be selected to produce a TEVG of this kind. Typical fabrications procedures consist of electrospinning (Soffer et al., 2008), freeze-drying (Engbers-Buijtenhuijs et al., 2006; Zhang et al., 2006), and mold casting (Boccafoschi et al., 2007; Schutte et al., 2010) (Figure 1C). Electrospun meshes of gelatin (Elsayed et al., 2016) have been used, usually in combination with a polymeric structure, to improve the surface conjugation with cells, while silk-fibroin nanofibers tubes have been used alone (Marelli et al., 2010) or as a support matrix for coating hydrogel such as collagen (Marelli et al., 2012) and gelatin (Marcolin et al., 2017). The feasibility of the approach has been proven *in vitro*, with the tubular scaffold showing encouraging mechanical properties (burst pressure of 1075 ± 444 mmHg) (Marcolin et al., 2017).

Following the Weinberg and Bell study in 1986, most of the studies focusing on natural materials have used a gel-based approach. This consists of casting a mixture of the desired gel and cell suspension in a tubular mold made of a polypropylene tube. The first period of incubation and cell growth is followed by a period of maturation in dynamic conditions to confer properties of a vascular tissue (Weinberg and Bell, 1986). Tranquillo et al. achieved the fabrication of a tubular structure using a fibrin gel with human dermal fibroblasts, but the burst pressure after 3 weeks was still far below (543 mmHg) the SV values (Huynh and Tranquillo, 2010). Natural gels, based on collagen and fibrin, have been evaluated as a possible artificial arterial conduit in animal models. The above mentioned fibrin-based structure with fibroblasts was implanted into the femoral artery of sheep. Cyclic deformation in addition to pulsatile flow conditioning improved mechanical properties of the graft (Syedain et al., 2011). To further improve the mechanical properties of the TEVG, a suspension of ovine dermal fibroblasts were added to the fibrin and the gelled structure was cultured for a total period of 5 weeks (the first 2 in static conditions and the remaining 3 under pulsatile flow stimulation) (Syedain et al., 2014). At the end of the conditioning period, the structure was decellularized. This process led to obtaining a TEVG with around 4200 mmHg burst pressure, able to remain patent for 24 weeks and with a concentration of collagen and elastin close to the natural values (Syedain et al., 2014). In another study, VSMCs were used to colonialize the fibrin gel and ECs were seeded on to the luminal side before implantation into an ovine model (Swartz, 2004; Liu et al., 2007). This graft showed good results in terms of integration with the native vessel but, on the other hand, it displayed poor mechanical properties.

Silk fibroin-based materials were considered as an alternative (Enomoto et al., 2010) and Kaplan et al. implanted a graft of this kind in the abdominal aorta of rats (Lovett et al., 2010). No thrombosis was seen and implants remained patent, with the absence of occlusion or ischemia detected at the 1 month follow-up.

Collagen-based vascular grafts were recently tested *in vivo* and assessed for the feasibility of the system to hold microsurgical sutures. Dehydration of crosslinked collagen allowed to create a small-diameter TEVG (diameter ≤ 1 mm) with a burst pressure of approximately 1313 mmHg, compliance of 1.7%/100 mmHg (comparable with mammalian vein), but the strength of the anastomosis at the interface between the rat femoral artery and TEVG was still lower than the one between two portions of explanted rat femoral artery (Li et al., 2017). Natural polymers are acknowledged to be valid alternatives in the production of small diameter TEVGs, due to their higher biocompatibility and capability to remodel *in vivo*. Nonetheless, natural polymers generally offer reduced mechanical strength compared to their synthetic counterparts and can be more susceptible to degradation, which, if not carefully controlled, may lead to rupture and aneurysm formation.

##### Decellularized natural matrices

The mismatch of mechanical properties, in terms of strength, elasticity, long-term resistance, and fatigue, between the fabricated scaffold and the native vessel led to development of grafts with a structure more similar to the biological ones, but available as an off-the-shelf product. This need was reflected by further attempts to employ decellularization of tissues harvested from allogenic or xenogeneic sources (Figure 1). The elimination of cells is needed to avoid an immunological reaction from the recipient, but agents employed to this scope should have properties allowing preservation of the structure and function of the ECM. These techniques typically use detergents, like sodium dodecyl sulfate, octylglucoside, sodium deoxycholate, and enzymes, like dispase II, nucleases, phospholipase, and thermolysin, often in combination with mechanical and physical methods to accelerate the process (Crapo et al., 2011).

The approach based on natural matrices to obviate the problems associated with autologous grafts, led to the commercialization of a variety of decellularized products, such as Procol (Hancock Jaffe Laboratories Inc., Irvine, CA), the SG 100 SynerGraft® vascular graft, (CryoLife, Kennesaw, Georgia, USA) and the already mentioned Artegraft (North Brunswick, NJ). To improve the properties of these grafts, autologous ECs and VSMCs obtained from differentiation of bone marrow-derived cells were seeded in decellularized matrices and then tested in ovine (Tillman et al., 2012) and canine models (Cho et al., 2005).

Decellularized vascular grafts derived from bovine have been widely experimented in clinical trials (Katzman et al., 2005; Chemla and Morsy, 2009) in which their performance was compared with the classical PTFE graft in arteriovenous fistula (AVF) and bypass procedures (Butler et al., 1977). Despite the investments, the grafts maintained high costs, showed low patency and multiple cases of immunogenic response due to the inefficacy in the decellularization process. Sumitran-Holgersson et al. performed the first human study on a single pediatric patient (Table 1), using a decellularized human iliac vein seeded with autologous cells (Olausson et al., 2012). The outcomes were promising, with patency up to 2 years even if applied in a low-flow district. A recent long-term study (2002–2017) showed the application of a bovine carotid artery graft (Artegraft) in lower extremity bypass surgery (Lindsey et al., 2017). Follow-up of 5 years of primary endpoints showed positive results for patency (66.7%) and salvage limb rates (81% of treated cases) (Table 1).

Among the non-vascular tissues, swine or bovine pericardium have been tested in the past, but porcine small intestinal submucosa (SIS) was the first to be assessed as a valid tissue source (Sandusky et al., 1995). SIS, used as an arteriovenous shunt in a sheep model, showed burst pressure of around 1200 mmHg and the cellularization of the graft improved the anticoagulation properties with a lower rate of platelet aggregate formation (Peng et al., 2011). The decellularization process has many disadvantages. In fact, the main cause of failure is related to immune response induced by leftovers of foreign cellular material. Although the biological origin of the tissue reduced the gap between the properties of the native vessel and the built graft, a persistent limitation consists of the divergent behavior under long-term stress. This difference leads to failure of the graft and possible creation of an aneurysm. In addition, decellularized grafts originating from non-vascular tissue, even if more reliable, are unsuitable for applications in which the scaffold has the necessity to adapt and grow with the patient, as in the case of correction of congenital vascular defects. In these patients, atherosclerotic and fibrotic remodeling and calcification are the most common consequences of poor integration of the graft with the surrounding tissue, resulting in stenosis and graft failure and requiring multiple interventions for substitution (Shetty et al., 2009; Gössl et al., 2012; Woo et al., 2016).

#### Hybrid scaffolds

Natural and synthetic polymers can be used together to create a composite scaffold in order to improve the characteristics that each category possess on its own. In recent years, initially positive results led to investment in this approach. A three-layered TEVG has been fabricated overlapping nanofibers of PCL, collagen, and PLLA (Haghjooy Javanmard et al., 2016). PCL has also been used blended with collagen (Tillman et al., 2009; Bertram et al., 2017), gelatin (Jiang et al., 2017) and elastin (Wise et al., 2011) to improve surface adhesion features, while a PLCL porous scaffold was coated with nanofibers of silk fibroin (Henry et al., 2017) or alternatively with a layer of hMSCs/ECs (Ahn et al., 2015; Pangesty et al., 2017).

PEG-fibrin hydrogel, with murine smooth muscle progenitor cells, was reinforced with an inner layer of electrospun PU fibers (McMahon et al., 2011). *in vivo* experiments with composite TEVGs were performed in the last decade demonstrating the feasibility of the hybrid approach. Murine models have been used to evaluate TEVGs composed of nanofibers of PCL blended with spider silk and chitosan (Zhao et al., 2013), and the scaffold showed maintenance of the patency for up to 8 weeks. A more recent experiment involved a decellularized rat aortic vessel in which the lumen was coated with heparin. Aiming at preventing the vessel weakening and consequent aneurysm formation, the decellularized structure was externally reinforced with PCL (Gong et al., 2016). This hybrid scaffold was easily sutured and displayed improved mechanical properties compared to rat autografts, with a burst pressure of 2060 mmHg and patency rate of 100% after 10 weeks implantation. In a large animal model, Poly(L/D)lactide (P(L/DL)LA) coated with fibrin gel was used as an interposed carotid artery graft in sheep (Koch et al., 2010). Autologous ECs, VSMCs, and fibroblasts were encapsulated in the fibrin gel and cast around the synthetic polymer before the bioreactor conditioning for 21 days to allow for the maturation of the cells. The TEVG was then implanted and after 6 months it showed the absence of thrombus and full patency. A study was recently performed on a large animal, grafting a PCL/collagen scaffold seeded with autologous ECs and VSMCs as arterial interposition. Computed tomography (CT) scan and ultrasonography showed no stenosis and structural integrity of the TEVG at 6 months follow-up (Ju et al., 2017). Though the hybrid approach offers an opportunity of exploiting the qualities of natural and synthetic polymers, a typical drawback is a need for long conditioning and the requirement of high technological skills during the manufacturing process.

### Self-assembled TEVG

**Figure 2:**
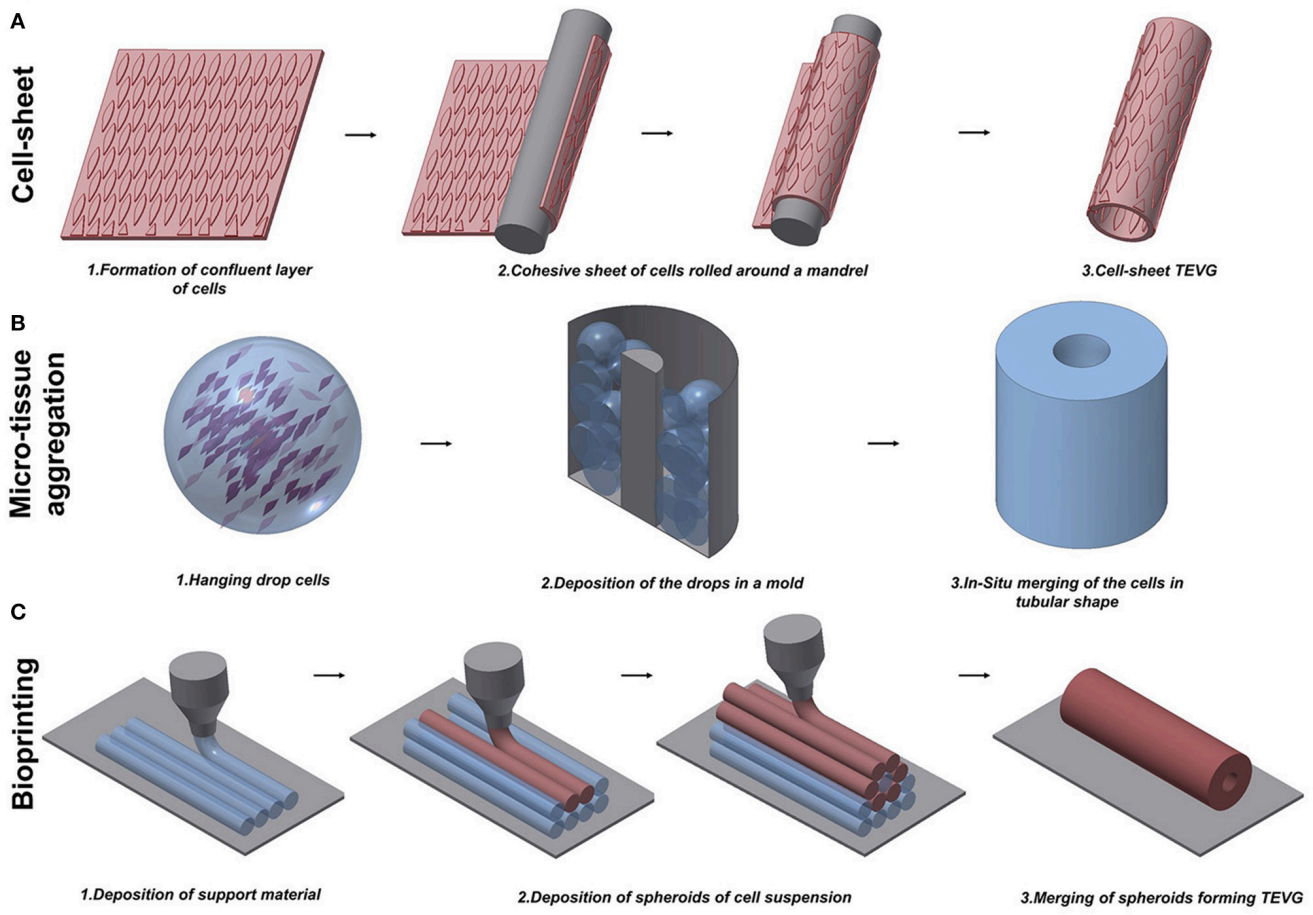
Schematic representation of techniques to manufacture scaffold-free vascular grafts. **(A)** Cell-sheet TEVG approach involves the use of a dense and cohesive sheet of cells to create a tubular structure. The sheet is rolled around a mandrel and matured under dynamic conditions to develop vessel-like properties. **(B)** Hanging drop cells are accurately positioned into a mold in the microtissue aggregation approach **(B)**. The production of ECM allows the merging of the aggregates in a complex structure. **(C)** Bioprinting exploits the extrusion of support material to increase the flexibility of the fabrication. An accurate design of support material and cells extruded in spheroids allows the creation of tubular structures.

Despite the improvements achieved in fabricating TEVGs based on scaffolds, some research groups believed that scaffolds would force the cells to grow in an unnatural assembly, and therefore decided to test the tissue engineered self-assembled (TESA) approach. Self-assembled TEVGs are based on the concept that cells, placed in a 3D environment and with the right stimuli, would be able to organize themselves in a complex tissue. Currently, three main strategies have been investigated: cell-sheet assembly, micro-tissue aggregation, and cell printing (Figure 2). Different cell sources have been considered suitable to generate a cell-sheet tissue engineered graft. Human adipose-derived stromal cells (Vallières et al., 2015) and dermal or SV fibroblasts (Bourget et al., 2012) were used to create a scaffold-free graft and, after *in vitro* characterization, displayed good mechanical properties and the ability to produce ECM proteins, including collagen type I, III, and IV, laminin and fibronectin, which are necessary to give structural support to the vessel. The development of this novel scaffold-free approach finds its origin in the success of the first trial by the pioneer L'Heureux. In 1998, L'Heureux et al. first documented the feasibility of implanting a cell-sheet graft in a canine model (L'Heureux et al., 1998). The technique consisted of peeling off a confluent culture of VSMCs and fibroblasts and carefully shaping the cell sheet with all the released ECM into a tubular structure (Figure 2A). Conditioning in a bioreactor represents a crucial step to allow the maturation of the cells by fusion of the wrapped layers in a unique vascular structure. After the extended conditioning (8 weeks) under dynamic conditions, the graft showed a burst pressure of around 2600 mmHg, which is higher than that of SV. Relevant to the physiological maturation of the graft, the authors showed the production of ECM proteins typical of the vasculature and the recruitment of ECs onto the luminal surface. Nonetheless, at 7 days post-implantation, the grafts exhibited bleeding and detachment of the layers leading to the failure of the implant. To overcome these limitations, a new study to fabricate scaffold-free TEVGs was performed, involving only human fibroblasts and increasing the period in dynamic culture (L'Heureux et al., 2006). The new grafts showed greater performances from the point of view of mechanical properties (burst pressure of 3468 mmHg) and were used as an arterial interposition in primate models. The grafts were shown to be resistant, did not form aneurysms and were fully patent after 8 weeks. Overall, the main limitation of the technique is the time to produce the graft which, considering the duration between the formation of a confluent sheet and the fusion throughout the bioreactor conditioning, was assessed to be around 28 weeks (L'Heureux et al., 2006). Scaffold-free grafts, containing autologous mesenchymal stem cells (MSCs), were tested by Zhao et al. during pre-clinical studies in a rabbit model (Zhao et al., 2012). The TEVG, used to replace a section of carotid artery 1 cm in length, was fully patent and completely endothelialized after the 4 weeks from implantation. An assessment of histology showed a full integration of the cell-sheet graft and a more complex remodeling in the laminated structure after the period of implantation.

In the meantime, the scaffold-free engineered graft developed by L'Heureux et al., patented with the name of Cytograft (Cytograft Tissue Engineering, Inc., Novato, CA), was used at the beginning of the 2000s in a clinical trial involving 10 patients that suffered from end-stage renal disease (McAllister et al., 2009). Fibroblasts obtained from biopsies of the patient were used to create the autologous TEVG. The fabrication procedure, illustrated above, was completed with the devitalization of the luminal side of the graft, and subsequent seeding of autologous ECs. The period of time required to produce these grafts turned out to be around 7.5 months. Excluding one patient that died for a cause not related to the graft, the other 3 failures of the self-assembled blood vessel were related to dilatation, thrombosis, and aneurysms. After the 20 month trial, the overall results of the study were promising. Grafts had 78% patency rate at the time point of 1 month and 60% at 6 months. In this study, McAllister et al. demonstrated the feasibility of long distance graft delivery after fabrication in insulated and sealed conditions for long distance (McAllister et al., 2009). L'Heureux's group further improved the production of an off-the-shelf graft attempting to define a procedure to store the graft after the manufacturing process. The cell-sheet based scaffolds were devitalized to be frozen and at the moment in which the patient required the implantation they were thawed, rehydrated and autologous ECs were finally seeded on the luminal side (Wystrychowski et al., 2011). The new allogenic Lifeline™ (Cytograft Tissue Engineering, Inc.), without the seeding of the autologous ECs, was also used in patients as shunts for hemodialysis (Table 1). The mechanical properties of the graft were not affected by the thawing procedure and no immune response was detected (Wystrychowski et al., 2014).

Microtissue aggregation represents a variation among the TESA strategies, in which, to circumvent mechanical stress, the cells are not peeled from the culture surface and the post-culturing manipulation to shape the graft is avoided using a growing template. In this approach, a temperature–responsive poly(N-isopropylacrylamide) is used as culturing surface, allowing the easy detachment of the cell aggregate when the culture is confluent (Asakawa et al., 2010). Alternatively, high density hanging drop cells are deposited in the mold for *in situ* merging by secretion of ECM, representing building blocks for vascular-like structures (Marga et al., 2012). Kelm et al. obtained a tubular structure by the aggregation of human artery-derived fibroblasts and HUVECs (Kelm et al., 2010) (Figure 2B). Fourteen days of conditioning with dynamic pulsatile flow allowed the fusion of the multiple blocks in a unique tissue with layered tissue formation.

One typical constraint of the TESA technique is the limited shape that the graft can assume. In fact, the inability of the cell aggregation to self-sustain, forced the researcher to keep the geometry as simple as possible. The possibility to rectify this limitation was recently proposed by the use of Bioprinting, which may have the refined capability to build a patient-specific arterial vessel (Figure 2C). Bioprinting is currently used to create constructs for growth factor delivery (Gao et al., 2015), *in vitro* microvacularized constructs (Kamei et al., 2006; Cui and Boland, 2009; Miller et al., 2012; Bertassoni et al., 2014; Kolesky et al., 2014; Gao et al., 2015) and myocardial patches (Gaebel et al., 2011; Gaetani et al., 2012, 2015). Some studies have focused on providing a proof-of-concept for manufacturing vascular structures with the intention to be used as small-diameter TEVGs (Borovjagin et al., 2017; Duan, 2017). A novel approach exploited the concept of self-assembly through the fusion of cell spheroids forming a unique tubular structure (Mironov et al., 2009). Norette et al. performed an initial study attempting to bioprint a complex vascular tree (Norotte, 2009). The high flexibility of this approach allowed the fabrication of tubular structures by aggregation of spheroids, with connected branches of accurate diameter and wall thickness. On the other hand, this strategy showed limitations in terms of sterility and its time-consuming nature. Maintaining the integrity of the environment during the assembling of the vascular tree is challenging, and a minimum of 7 days is required to allow for the fusion of the spheroids on to the tubular surface. To overcome some of these disadvantages, Norette et al. reduced the complexity of the system and succeeded in the creation of a tubular structure by deposition of human umbilical vein VSMCs and skin fibroblasts (Norotte, 2009). Additionally, mouse embryonic fibroblasts have been used by Kucukgul et al. to fabricate a scaffold-free arterial construct (Kucukgul et al., 2015). A bioprinted vascular graft suitable for implantation and delivery to any large *in vivo* model or clinical trial has not been developed, mainly due to the lack of mechanical properties and the long period required to produce a stable structure.

## Conclusions

The necessity to find an alternative to autologous vascular grafts led to the development of TEVG, which, exploiting the combination of multiple approaches, holds promise to match the minimal requirement of current autologous vessels. Many improvements have been awarded in the recent past and results from *in vivo* experiments showed encouraging outcomes. Nevertheless, initial clinical trials did not always confirm the experimental findings, thus suggesting there is still room for improvements in the translational process.

Failure of TEVG could occur at different time points after the implantation and, accordingly, can be classified as early, midterm and late failures (Pashneh-Tala et al., 2015). Acute thrombosis is the main cause of early failure (within 3 months after implantation) and it is a coagulation reaction driven by platelet adhesion on collagen, which in the native vasculature is avoided by the anti-thrombotic properties of the endothelium. Acellular and decellularized TEVGs are mostly affected by this issue. Biodegradable TEVGs, cellularized or based on hybrid approach, shielding the scaffold lumen from the bloodstream, show a reduction in thrombus events. Multiple solutions have been explored, combining different synthetic and natural materials and performing chemical functionalization of the surface to improve anti-thrombogenic properties (Seifu et al., 2013; Tara et al., 2014). Furthermore, biodegradable polymers offer the possibility for the cells to colonialize the porous structure and thereby stimulate the production of ECM proteins. On the other hand, if the properties of the polymers are not well tuned, the intimal thickening can reduce the patency caused by the excessive migration and proliferation of cells. Lumen occlusion due to anastomotic intimal hyperplasia typically characterizes the midterm failures (from 3 months to 2 years). Late term failure is associated, instead, to recurrent atherosclerotic disease and is a common problem with all the current approaches. It is mainly due to the loss in consistency of the graft or poor *in vivo* integration.

So far, decellularized native tissues are the most successful *in vivo* approach, although post-implantation thrombus events are the main limitation for low long-term patency. Moreover, decellularization is still a topic of debate because the incompleteness of the process may lead to an immunogenic reaction by the recipient, whereas an excessive chemical treatment can provoke the loss of mechanical properties and aneurysmal dilatation (Shojaee and Bashur, 2017).

On the other hand, recent improvements in biodegradable TEVG production, offering a wider range of physical properties and the capability to remodel *in vivo*, might represent a potential solution to generate a valid TEVG. New microfabrication technologies allow patient-specific manufacturing of the TEVG, eliminating the dimension mismatch at the anastomosis site. This approach is particularly suitable for correction of congenital related diseases. Nevertheless, patency rate is still drastically lower than autologous grafts, with *in vivo* animal experiments no longer than 2 years.

For the category of cellularized TEVGs, the cell source represents a much discussed topic. As described in this review, an extensive variety of cells have been tested, both to build the core of the graft (in the case of a scaffold-free approach) and to cellularize the scaffold in the pre-implantation stage. Autologous mature vascular cells, like VSMCs, ECs, and fibroblasts, have been used to cellularize TEVGs with success. Here, the main limitation is represented by the extraction from a patient biopsy, thereby leading to insufficient expansion (G et al., 2015). Adult stem cells, such as BM-MSCs have been used in consideration of their high proliferative property. The time-consuming process of fabrication, cellularization or chemical treatment is often not compatible with the urgent need of patients suffering severe pathology. This can only be solved through the adoption of an off-the-shelf TEVGs. In this approach, standardization of the fabrication process, preservation of sterile conditions and delivery represent the main challenges. However, the potential of having a ready to use graft, with advantages for patients and clinicians, is becoming an attracting prospective.

Owing to the growing target-population for tissue engineering technologies/products under development for cardiac and vascular indications, as well as the major healthcare costs associated with existing treatments, the potential financial figure for these products is in the range of multibillion-dollar volume. In the U.S. alone, the total potential market for tissue engineering and cell transplantation technologies is expected to exceed USD 22.8 billion in the year 2019. Furthermore, the global vascular graft market is expected to reach USD 3,626 million by 2022, according to a new study by Grand View Research, Inc. There is a positive prospect that rising healthcare expenditure, favorable reimbursement policies, and technological breakthroughs will boost growth in the vascular graft market over the next 10 years. At present, TEVGs contribute a minimal part of these financial figures, however, a number of positive factors indicate that this new technology will be successfully translated from research into medical practice.

## Author contributions

MC: searched the literature and drafted the manuscript; PM: critically revised the work.

### Conflict of interest statement

The authors declare that the research was conducted in the absence of any commercial or financial relationships that could be construed as a potential conflict of interest.
